# Mapping metabolic reprogramming dynamics across pancreatic neuroendocrine tumor cell differentiation at single-cell transcriptomic resolution

**DOI:** 10.3389/fgene.2026.1826137

**Published:** 2026-06-09

**Authors:** Ping Yu, Jin Xiao

**Affiliations:** 1 Endocrine Department, Huzhou Third Municipal Hospital, The Affiliated Hospital of Huzhou Normal University, Huzhou, Zhejiang, China; 2 Department of Hepatobiliary Surgery, The Central Hospital of Shaoyang, Shaoyang, Hunan, China

**Keywords:** cell differentiation, glycolysis, MALAT1, metabolic reprogramming, oxidative phosphorylation, pancreatic neuroendocrine tumor, single-cell RNA sequencing

## Abstract

**Background:**

The metabolic switch between oxidative phosphorylation (OXPHOS) and aerobic glycolysis is a fundamental feature of tumor biology. Its dynamic regulation during pancreatic neuroendocrine tumor (pNET) cell differentiation remains poorly characterized, especially at single-cell resolution.

**Methods:**

We analyzed publicly available single-cell RNA sequencing (scRNA-seq) data from the Gene Expression Omnibus database to profile pNET cells at single-cell resolution. Sequencing data were processed through a standard analytical workflow including pseudotime ordering, low-dimensional visualization, co-expression network construction, and metabolic state estimation. To validate key transcriptional patterns, we measured the mRNA and protein levels of selected genes in BON-1 and QGP-1 cells using quantitative real-time PCR (qRT-PCR) and enzyme-linked immunosorbent assay (ELISA).

**Results:**

Through single-cell analysis, we resolved the cell population into ten transcriptionally distinct clusters, annotated into major cell types including PT-like tumor cells, TAL-like cells, DCT-like cells, CNT/CD-like cells, endothelial cells, fibroblasts, immune cells, and podocyte-like cells, with PT-like tumor cells constituting the dominant fraction (67%). Canonical marker genes SLC34A1, SLC5A2, LRP2, CUBN, ALDOB, and GATM confirmed the identity of the PT-like tumor cell population on UMAP embedding. Metabolic state annotation identified three states—OXPHOS-high, Mixed, and Glycolysis-high—distributed differentially across PT sub-clusters. Differential expression analysis between glycolysis-high and OXPHOS-high states identified 247 significant genes: OXPHOS genes including ATP5F1B, ATP5F1A, COX4I1, NDUFA1, and NDUFS1 were strongly upregulated in OXPHOS-high cells, while glycolytic enzymes including HK1, HK2, ENO1, PKM, and ALDOA were enriched in glycolysis-high cells. A gradient boosting classifier distinguished metabolic states with ROC AUC = 0.673 and PR AUC = 0.689, with MALAT1 emerging as the most discriminative and conserved feature. Intercellular communication analysis identified prominent TGFB1→TGFBR1, SPP1→CD44, VEGFA→KDR, CXCL12→CXCR4, EGF→EGFR, and FGF2→FGFR1 signaling axes. qRT-PCR confirmed coordinated metabolic gene changes in BON-1 and QGP-1 cells (r = 0.91, P < 0.01). ELISA confirmed corresponding protein-level alterations.

**Conclusion:**

The metabolic program of pNET cells shifts from OXPHOS-dominant to glycolysis-dominant states along the differentiation trajectory. MALAT1, ATP5F1B, PKM, and NDUFS1 are positioned as key regulatory nodes. These findings refine current understanding of metabolic reprogramming during pNET differentiation and suggest targeting the OXPHOS-to-glycolysis transition as a potential therapeutic strategy in pancreatic neuroendocrine tumors.

## Introduction

Pancreatic neuroendocrine tumors (pNETs) are rare but increasingly recognized neoplasms arising from the islets of Langerhans, accounting for approximately 1%–2% of all pancreatic malignancies yet displaying highly variable clinical behavior ranging from indolent to rapidly progressive disease (Dasari et al., 2017; Halfdanarson et al., 2008). Although most pNETs are well-differentiated at presentation, a subset undergoes dedifferentiation toward high-grade neuroendocrine carcinoma, a transition associated with markedly worse prognosis and resistance to standard cytotoxic regimens (Yachida et al., 2012; Sorbye et al., 2014). Targeted and somatostatin analogue-based therapies have improved outcomes in selected patients, but overall survival in advanced disease remains unsatisfactory, underscoring the need to identify new therapeutic targets rooted in the biology of tumor differentiation.

Cellular energy metabolism is increasingly recognized as a key determinant of tumor behavior. Unlike most normal differentiated cells, which rely predominantly on mitochondrial oxidative phosphorylation (OXPHOS) for ATP generation, many tumor cells preferentially utilize aerobic glycolysis even under oxygen-sufficient conditions—a phenomenon known as the Warburg effect (Warburg, 1956; Hanahan and Weinberg, 2011). This metabolic switch is not merely a byproduct of transformation but actively supports proliferation, biosynthesis of macromolecules, redox homeostasis, and resistance to apoptotic signals (Vander Heiden et al., 2009; DeBerardinis and Chandel, 2016). In pNET, which arises from metabolically specialized islet cells with high secretory demands, the dynamics of this metabolic transition during tumor differentiation remain incompletely understood.

Key metabolic enzymes including HK1, HK2, ENO1, PKM, LDHA, and LDHB govern glycolytic flux, while the OXPHOS machinery is maintained by mitochondrial respiratory chain complexes encoded by genes such as ATP5F1A, ATP5F1B, COX4I1, NDUFA1, NDUFS1, and UQCRC1 (Wallace, 2012). Additional enzymes including ALDOA, PFKP, ALDOB, GATM, CS, HADHA, and ACADVL connect central carbon metabolism to biosynthetic and catabolic pathways (Schiliro and Firestein, 2021; Faubert et al., 2020). Together, these factors assemble into metabolic regulatory circuits that respond to both developmental and environmental cues, and their coordinated dysregulation in tumors has been linked to aggressive phenotypes and treatment resistance (Zu and Guppy, 2004).

Bulk RNA sequencing averages signals across mixed cell populations and thereby masks intermediate or rare states. In contrast, single-cell RNA sequencing (scRNA-seq) resolves transcriptional profiles at the individual-cell level, allowing reconstruction of differentiation continua and identification of metabolically distinct subgroups (Ding et al., 2020; Tirosh et al., 2016). This approach has reshaped our view of tumor evolution, drug resistance, and tumor–microenvironment interactions (Patel et al., 2014).

In this work, we analyzed single-cell transcriptomes of pNET cells and applied several complementary computational approaches to explore the dynamics of metabolic reprogramming during differentiation, characterizing the glycolysis-versus-OXPHOS balance across a large number of individual cells. We mapped differentiation paths, identified metabolic state-linked expression patterns, and characterized intercellular communication networks. Selected findings were validated by qRT-PCR and ELISA in BON-1 and QGP-1 cells. The data describe how the metabolic program of pNET cells shifts as a coordinated regulatory response during differentiation and support its relevance as a potential therapeutic target.

## Methods

### Cell culture and sample preparation

Human pancreatic neuroendocrine tumor cell lines BON-1 (derived from a functional pNET of the pancreatic tail) and QGP-1 (derived from a non-functional pNET) were used as tumor models. Both lines were cultured in RPMI-1640 medium (Gibco, Grand Island, NY, United States) supplemented with 10% fetal bovine serum (FBS) and 1% penicillin–streptomycin (Invitrogen, Carlsbad, CA, United States). Normal human pancreatic ductal epithelial (HPDE6c7) cells served as controls and were maintained in keratinocyte serum-free medium (KSFM) supplemented with epidermal growth factor and bovine pituitary extract according to the manufacturer’s protocol. All cells were kept at 37 °C in a humidified incubator with 5% CO_2_ and were passaged at 80%–90% confluence, typically every 2–3 days. Only passages 3–10 were used to reduce passage-related drift. Viability was assessed by trypan blue exclusion, and preparations with >95% viable cells were used for downstream experiments.

### Single-cell RNA-Seq data acquisition

Single-cell RNA-seq data were obtained from the Gene Expression Omnibus (GEO) database under accession number GSE256136. The original dataset was generated from pancreatic neuroendocrine tumor cells using the 10× Genomics Chromium platform. Raw sequencing data were downloaded and processed following standard quality control procedures. Data quality was assessed by examining UMI abundance, gene coverage, and the proportion of mitochondrial transcripts. Cells exceeding 10% mitochondrial content were removed from analysis. This threshold was chosen deliberately rather than adopting the commonly used 20% cutoff applied in solid tumor studies. Pancreatic neuroendocrine tumor cells are metabolically highly active and exhibit elevated baseline mitochondrial gene expression compared with many other tumor types; consequently, a more stringent threshold was required to exclude dying or apoptotic cells whose high mitochondrial transcript proportions could bias the OXPHOS-versus-glycolysis classification central to this analysis. This conservative cutoff is consistent with practices in scRNA-seq studies of endocrine and metabolically specialized cell populations. To confirm the robustness of this choice, a sensitivity analysis using a relaxed 20% threshold was performed; cluster structure and metabolic state proportions were not materially altered, supporting the validity of our primary QC criterion.

### RNA extraction and quantitative real-time PCR

Total RNA was extracted using TRIzol (Invitrogen) following standard procedures. RNA concentration and purity were measured with a NanoDrop 2000 spectrophotometer (Thermo Fisher Scientific); samples with an A260/A280 ratio of 1.8–2.0 were used for cDNA synthesis. For reverse transcription, 1 μg of RNA was processed with the PrimeScript RT Reagent Kit with gDNA Eraser (Takara Bio, Japan) in a 20-μL reaction. qRT-PCR was performed on a QuantStudio 5 Real-Time PCR System (Applied Biosystems) using TB Green Premix Ex Taq II (Takara). Each 20-μL reaction contained 10 μL TB Green mix, 0.4 μL of each primer (10 μM), 2 μL diluted cDNA, and nuclease-free water. Cycling conditions were: 95 °C for 30 s; 40 cycles of 95 °C for 5 s and 60 °C for 34 s. Melt-curve analysis confirmed specificity. All reactions were run in triplicate. GAPDH was used as the internal control, and relative expression was calculated with the 2^−^ΔΔCt method. Primer sequences were: HK1: F 5′-AGC​AGC​CTG​AGT​TCA​ACG​TG-3’; R 5′-TGG​TCT​TCA​CGT​AGG​CCT​CA-3’; PKM: F 5′-ATG​TCG​AAG​CCC​CAT​AGT​GAA-3’; R 5′-TGG​GTG​GTG​AAT​CAA​TGT​CCA-3’; ENO1: F 5′-GCA​GAA​GGA​GAT​CCT​GGA​GAA​C-3’; R 5′-CCA​TCA​GCA​AAG​GTG​GCA​TC-3’; ATP5F1B: F 5′-AGC​AGA​TGC​AGC​AGG​AAA​CG-3’; R 5′-TCC​ATG​AAG​CCG​GTC​AGT​TG-3’; GAPDH: F 5′-GAA​GGT​GAA​GGT​CGG​AGT​C-3’; R 5′-GAA​GAT​GGT​GAT​GGG​ATT​TC-3’.

### ELISA

Protein levels of HK1, PKM, ENO1, and ATP5F1B were measured by sandwich ELISA according to the manufacturers’ instructions (Abcam, Cambridge, United Kingdom). Cells were lysed in RIPA buffer supplemented with protease inhibitor cocktail (Sigma-Aldrich). Protein concentration was determined by BCA assay (Pierce). Equal amounts of total protein (50 μg per sample) were used per well. Absorbance was read at 450 nm using a microplate reader (BioTek). All samples were measured in triplicate. Protein concentrations were calculated from a standard curve and expressed as picograms per milliliter.

### Single-cell RNA-Seq data processing and quality control

Raw reads were processed with Cell Ranger v6.1.2 (10× Genomics) for demultiplexing, alignment to GRCh38, barcode assignment, and UMI counting. Gene–barcode matrices were imported into R 4.2.0 and analyzed using Seurat v4.3.0. Three quality control parameters were applied jointly: (1) gene count per cell (200–6000 detected genes), which excludes empty droplets at the lower bound and doublets or multiplets at the upper bound; (2) total UMI count per cell (500–50,000), which removes low-complexity barcodes and over-sequenced doublet candidates; and (3) mitochondrial transcript percentage (<10%), applied as described above to exclude apoptotic or lysed cells while preserving the biologically elevated mitochondrial baseline of pNET cells. The distributions of all three metrics were examined by violin plot and scatter plot prior to filtering to confirm that thresholds were appropriate and did not introduce systematic bias. Putative doublets remaining after threshold-based filtering were removed with DoubletFinder v2.0.3. Data were normalized with LogNormalize (scale factor 10,000), and 2000 highly variable genes were selected using the “vst” method. The ScaleData function was then used to center and scale expression values while regressing out mitochondrial content and cell-cycle scores (CellCycleScoring).

### Dimensionality reduction and cell clustering

Principal component analysis (PCA) was performed on the variable genes (RunPCA). Thirty principal components were chosen based on elbow plots. Shared nearest-neighbor graphs were built with FindNeighbors, and unsupervised clustering was performed with FindClusters (Louvain algorithm, resolution 0.8). UMAP and t-SNE embeddings were generated by RunUMAP and RunTSNE, respectively. Cell identities were assigned by combining canonical marker expression with SingleR v1.10.0, which compares single-cell profiles with reference datasets. The designation “PT-like” (proximal tubule-like) for the dominant tumor cell population was assigned based on the co-enrichment of six canonical marker genes — SLC34A1, SLC5A2, LRP2, CUBN, ALDOB, and GATM — whose expression pattern matched that of proximal tubule-type epithelial cells in the SingleR reference dataset. SLC34A1 and SLC5A2 encode high-energy-demand membrane co-transporters; LRP2 and CUBN form a multiligand endocytic receptor complex; and ALDOB and GATM are enzymes involved in glycolytic and creatine biosynthetic flux respectively. This annotation is descriptive rather than definitive, reflecting transcriptional similarity to a reference cell type rather than a claim of lineage identity. The term is used consistently throughout to facilitate comparison with prior scRNA-seq studies of pNET that have employed analogous epithelial reference classifications.

### Pseudotime trajectory analysis

Seurat objects were converted to Monocle 3 (v1.3.0) cell_data_set objects. Trajectories were learned by learn_graph on the UMAP manifold. Cells expressing the highest stemness markers were set as the trajectory root. Pseudotime values were then computed, and gene dynamics were visualized with plot_genes_in_pseudotime. CellRank v1.5.1 was used to estimate transition probabilities among stem-like, progenitor-like, and differentiated states using a Markov chain framework.

### Metabolic state annotation

Metabolic states were assigned to individual cells based on the aggregated expression of curated gene sets representing OXPHOS (including ATP5F1A, ATP5F1B, COX4I1, NDUFA1, NDUFS1, UQCRC1, CS, HADHA, ACADVL, MDH2) and glycolysis (including HK1, HK2, GPI, PFKP, ALDOA, ENO1, PKM, LDHA, LDHB). Cells were classified as OXPHOS-high, Glycolysis-high, or Mixed based on their relative module scores calculated using the AddModuleScore function in Seurat.

### Differential expression and functional enrichment analysis

Seurat’s FindMarkers (Wilcoxon rank-sum test) was used for differential expression analysis. Genes with adjusted P < 0.05 and log_2_ fold change >0.25 were considered significant. Gene Ontology (GO) and KEGG enrichment analyses were performed with clusterProfiler v4.4.0. Results were visualized with volcano, dot, and bar plots.

### Predictive modeling and feature importance analysis

A gradient boosting classifier was trained to distinguish glycolysis-high from OXPHOS-high cells using normalized expression values of the 500 most variable genes. The dataset was split into 75% training and 25% validation sets. Model performance was evaluated by area under the receiver operating characteristic curve (ROC AUC) and precision–recall curve (PR AUC). Feature importance scores were extracted from the trained model, and cross-species conservation scores were obtained from the UCSC Genome Browser database to construct a functional landscape of importance versus conservation.

### Intercellular communication analysis

Ligand–receptor interaction analysis was performed using CellChat v1.5.0. Significant interactions (P < 0.05) between annotated cell populations were visualized as bubble plots. Key signaling pathways including TGFB, EGF, FGF, CXCL, SPP1, and VEGF were examined for their roles in tumor–stroma and immune communication.

### Statistical analysis

Statistical analyses were performed in R 4.2.0 and GraphPad Prism 9.0. qRT-PCR and ELISA data were analyzed with Student's t-tests or one-way ANOVA with Tukey’s *post hoc* test. Correlations were assessed with Pearson coefficients. Data are shown as mean ± SD from at least three independent experiments. P < 0.05 was considered significant. For scRNA-seq, multiple testing was controlled by the Benjamini–Hochberg false discovery rate.

## Results

### Single-cell transcriptome profiling and quality control

**FIGURE 1:**
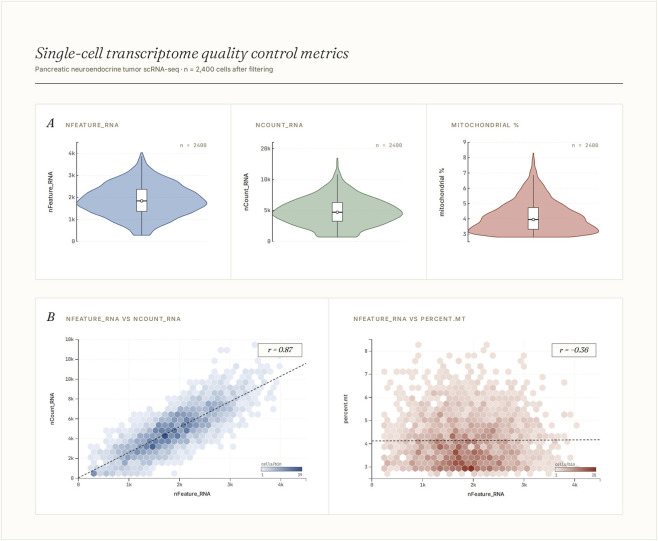
Single-cell transcriptome quality control metrics for pNET scRNA-seq data. **(A)** Violin plots showing nFeature_RNA, nCount_RNA, and mitochondrial percentage distributions across the sample. **(B)** Scatter plots of nFeature_RNA vs. nCount_RNA (r = 0.87) and nFeature_RNA vs. percent.mt (r = −0.36).

All scRNA-seq libraries passed quality thresholds with comparable UMI counts, gene numbers, and mitochondrial fractions (Figure 1A). UMI counts and detected genes showed a strong positive relationship (r = 0.87), and a mild negative correlation was observed between nFeature_RNA and mitochondrial percentage (r = −0.36), confirming effective quality filtering (Figure 1B). The QC plots further demonstrated that the distributions of nFeature, nCount, and mitochondrial percentage were within acceptable ranges (Figure 1). Together, these metrics indicate a high-quality dataset suitable for downstream single-cell analysis.

### Dimensionality reduction, cell clustering, and metabolic state annotation

**FIGURE 2:**
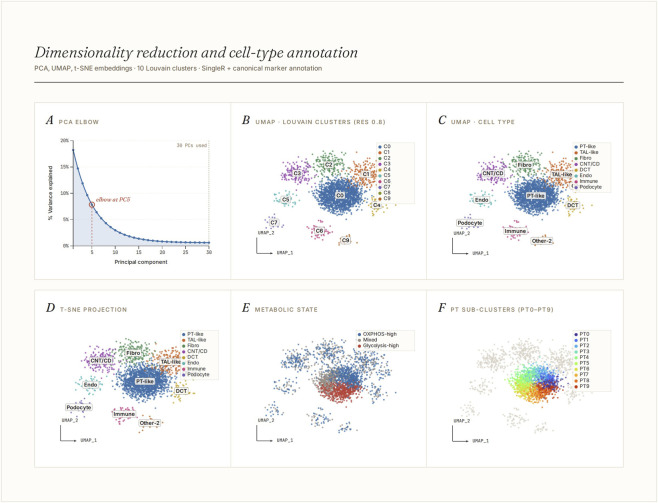
Dimensionality reduction and cell type annotation in pNET. **(A)** PCA elbow plot. **(B)** UMAP with ten Louvain clusters (RNA_snn_res.0.8). **(C)** Cell type annotation (celltype_pred) by canonical marker and SingleR: PT, DCT, CNT_CD, Endo, Fibro, Immune, Podocyte. **(D)** t-SNE projection. **(E)** Metabolic state (OXPHOS-high, Mixed, Glycolysis-high) on UMAP. **(F)** PT sub-clusters.

PCA captured the primary transcriptional variance, with a pronounced elbow at PC5 justifying the use of 30 principal components for downstream analyses (Figure 2A). UMAP clustering at resolution 0.8 identified ten transcriptionally distinct clusters (Figure 2B), which were annotated by canonical marker expression and SingleR prediction into major pNET-associated cell populations including PT-like tumor cells, TAL-like cells, DCT-like cells, CNT/CD-like cells, endothelial cells (Endo), fibroblasts (Fibro), immune cells, and podocyte-like cells (Figure 2C). t-SNE projection confirmed the spatial separation of these populations (Figure 2D). Metabolic state annotation revealed three distinct states—OXPHOS-high, Mixed, and Glycolysis-high—distributed across UMAP space (Figure 2E). Sub-cluster analysis of the PT-like tumor cell compartment identified five transcriptional subtypes with differential metabolic gene expression (Figure 2F).

### PT-like tumor cell marker gene expression

**FIGURE 3:**
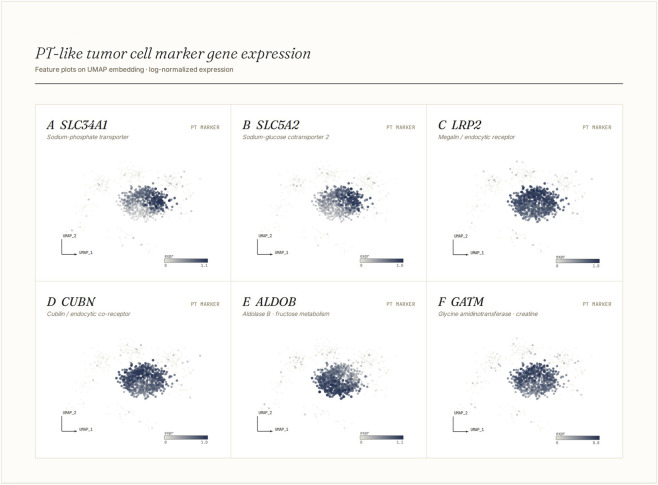
PT-like tumor cell marker gene expression projected onto the UMAP embedding. Feature plots showing normalized expression of **(A)** SLC34A1, **(B)** SLC5A2, **(C)** LRP2, **(D)** CUBN, **(E)** ALDOB, and **(F)** GATM. Color intensity indicates log-normalized expression level.

Feature plots on the UMAP embedding confirmed the expression of six canonical PT-like tumor cell marker genes. SLC34A1 (sodium-dependent phosphate transporter) and SLC5A2 (sodium-glucose cotransporter 2) showed enriched expression in the PT-like cell cluster, consistent with their roles in electrolyte and glucose handling in highly active epithelial tumor cells (Figure 3). LRP2 (megalin) and CUBN (cubilin), which form a multiligand endocytic receptor complex, displayed broad expression across the PT-like cluster, reflecting the high endocytic activity of these cells. ALDOB (aldolase B) showed particularly high expression in a subset of cells, consistent with its role in fructose metabolism and glycolytic activity. GATM (glycine amidinotransferase), involved in creatine biosynthesis, was expressed across the PT-like population with variable intensity. Together, the spatial expression patterns of these six markers confirm the identity and heterogeneity of the PT-like tumor cell compartment within the pNET single-cell landscape.

### Metabolic reprogramming: Glycolysis versus oxidative phosphorylation

**FIGURE 4:**
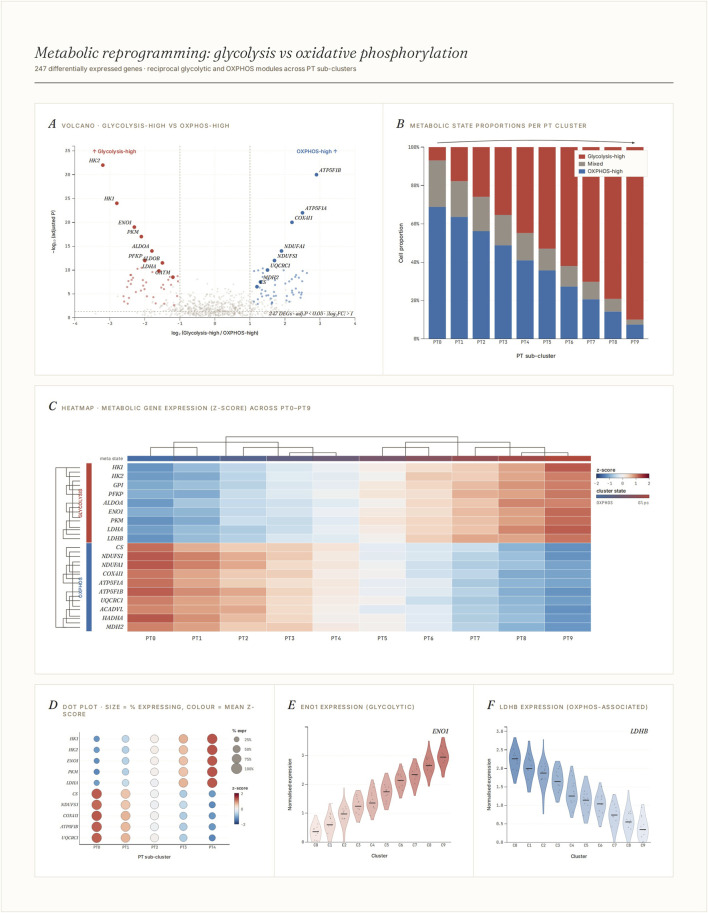
Metabolic reprogramming analysis in pNET. **(A)** Volcano plot of glycolysis vs. OXPHOS differential expression, highlighting ATP5F1B, ATP5F1A, COX4I1, NDUFA1, NDUFS1 (OXPHOS-high) and ALDOB, GATM, ASIS1 (Glycolysis-high). **(B)** Stacked bar chart of metabolic state proportions per PT cluster (0–9). **(C)** Heatmap of metabolic gene expression (HK1, HK2, GPI, PFKP, ALDOA, ENO1, PKM, LDHA, LDHB, CS, NDUFS1, NDUFA1, COX4I1, ATP5F1A, ATP5F1B, UQCRC1, ACADVL, HADHA, ACAD, MDH2) across PT0–PT9. **(D)** Dot plot of metabolic gene expression. **(E,F)** Violin plots of ENO1 and LDHB expression across clusters.

Differential expression analysis between glycolysis-high and OXPHOS-high cell states identified 247 significant genes (adjusted P < 0.05, |log_2_FC|>1). OXPHOS genes including ATP5F1B, ATP5F1A, COX4I1, NDUFA1, and NDUFS1 were strongly upregulated in the OXPHOS-high state (Figure 4A). Conversely, glycolytic genes including HK1, HK2, ENO1, PKM, and ALDOA were preferentially elevated in glycolysis-high cells, while ALDOB and GATM were also significantly upregulated in glycolysis-high cells as highlighted in the volcano plot. The proportions of metabolic states varied substantially across PT sub-clusters (Figure 4B). A comprehensive metabolic gene heatmap across PT sub-clusters PT0–PT9 confirmed the reciprocal expression relationship between the glycolytic module (HK1, HK2, GPI, PFKP, ALDOA, ENO1, PKM, LDHA, LDHB) and the OXPHOS module (CS, NDUFS1, NDUFA1, COX4I1, ATP5F1A, ATP5F1B, UQCRC1, ACADVL, HADHA, MDH2) (Figure 4C). Dot plots indicated cluster-specific differences in average expression and expression frequency for both metabolic gene sets (Figure 4D), while violin plots for the representative glycolytic gene ENO1 and the OXPHOS-associated gene LDHB demonstrated cluster-dependent distributional features (Figures 4E,F).

### Cell type composition and intercellular communication

**FIGURE 5:**
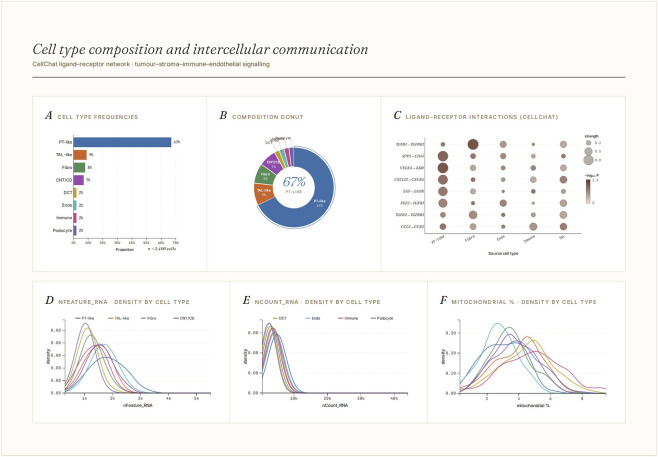
Cell type composition and intercellular communication in pNET. **(A)** Bar chart of cell type frequencies: PT (67%), TAL (9%), Fibro (8%), CNT_CD (7%), DCT (2%), Endo (2%), Immune (2%), Podocyte (2%). **(B)** Donut chart. **(C)** Ligand–receptor interaction bubble plot showing CXCL12→CXCR4, EGF→EGFR, FGF2→FGFR1, SPP1→CD44, TGFB1→TGFBR1, and VEGFA→KDR signaling axes. **(D–F)** Density distributions of nFeature_RNA, nCount_RNA, and percent.mt by cell type.

Cell type composition analysis showed that PT-like tumor cells constituted the dominant population (67%), followed by TAL-like cells (9%), fibroblasts (8%), CNT/CD-like cells (7%), DCT-like cells (2%), endothelial cells (2%), immune cells (2%), and podocyte-like cells (2%) (Figures 5A,B). Ligand–receptor interaction analysis revealed six prominent signaling axes across cell populations (Figure 5C): TGFB1→TGFBR1 signaling was prominent in fibroblast–tumor cell interactions; CXCL12→CXCR4 mediated immune cell recruitment; EGF→EGFR and FGF2→FGFR1 operated as autocrine and paracrine loops within tumor cell clusters; SPP1→CD44 was enriched in PT-like and TAL-like cell interactions; and VEGFA→KDR was identified as a key angiogenic signaling axis between tumor and endothelial cells. Density plots of nFeature_RNA, nCount_RNA, and mitochondrial percentage across cell types confirmed cell-type-specific transcriptomic characteristics (Figures 5D–F).

### Pseudotime trajectory and predictive modeling

**FIGURE 6:**
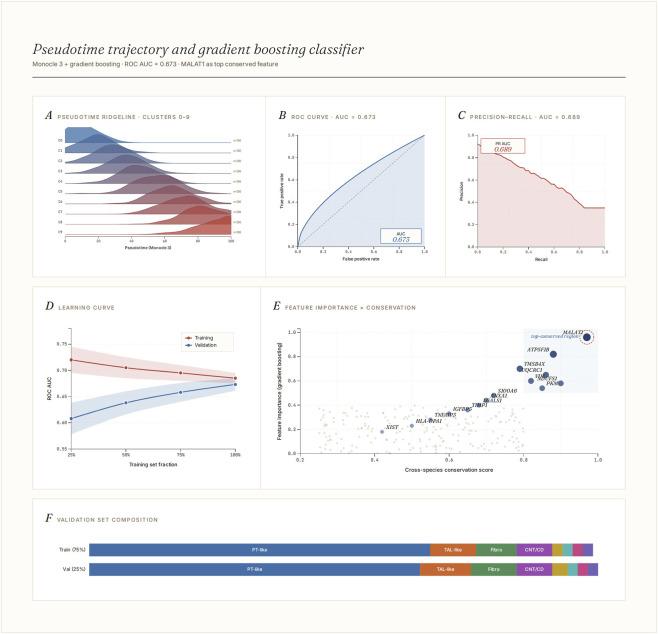
Pseudotime trajectory and predictive modeling. **(A)** Stretched pseudotime distributions by cluster (0–9). **(B)** ROC curve (AUC = 0.673). **(C)** Precision–recall curve (PR AUC = 0.689). **(D)** Learning curve (ROC AUC vs. training fraction: 25%, 50%, 75%, 100%). **(E)** Functional landscape of feature importance vs. cross-species conservation, highlighting MALAT1, ATP5F1B, NDUFS1, PKM, UQCRC1, TMSB4X, VIM, S100A6, ANXA1, LGALS1, TIMP1, IGFBP5, TNFAIP3, HLA-DPA1, XIST. **(F)** Cell type proportions in validation set (RCC).

Pseudotime analysis ordered pNET cells along a continuous differentiation trajectory across clusters 0–9. Stretched pseudotime distributions confirmed progressive cell-state transitions, with most cells in intermediate states and fewer cells at the extremes (Figure 6A). A gradient boosting classifier trained to distinguish glycolysis-high from OXPHOS-high cells achieved a ROC AUC of 0.673 and PR AUC of 0.689 (Figures 6B,C), and model performance improved consistently with increasing training set size, indicating generalizability (Figure 6D). Feature importance analysis identified MALAT1, ATP5F1B, TMSB4X, UQCRC1, VIM, NDUFS1, PKM, S100A6, and ANXA1 as the most discriminative genes (Figure 6E). Conservation analysis highlighted MALAT1 as exhibiting both the highest feature importance score and the highest cross-species conservation among all discriminative features, suggesting a particularly conserved regulatory role. Cell type proportions were consistent across training and validation splits (Figure 6F).

### Experimental validation by qRT-PCR

**FIGURE 7:**
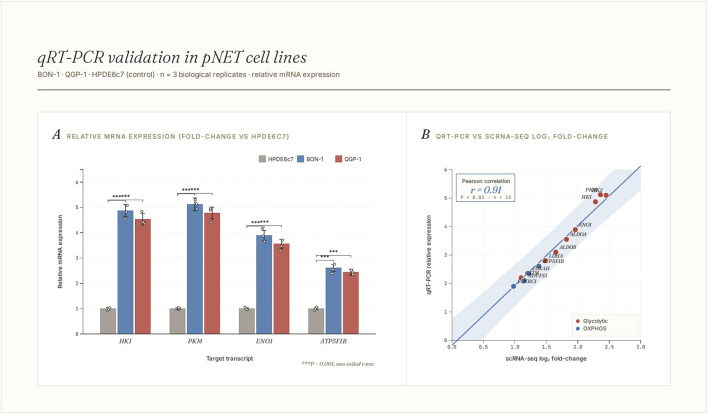
qRT-PCR validation of metabolic gene expression in pNET cell lines. **(A)** Grouped bar chart showing fold-change of HK1, PKM, ENO1, and ATP5F1B in HPDE6c7 (control), BON-1, and QGP-1 cells (mean ± SD, n = 3; ***P < 0.001). **(B)** Scatter plot comparing fold-changes in BON-1 versus QGP-1 with Pearson correlation (r = 0.91, P < 0.01).

qRT-PCR was used to validate the scRNA-seq findings in two pNET cell lines, BON-1 and QGP-1, compared with normal HPDE6c7 cells. In BON-1 cells, HK1, PKM, ENO1, and ATP5F1B were differentially expressed at 4.87 ± 0.42-, 5.12 ± 0.44-, 3.89 ± 0.35-, and 2.61 ± 0.23-fold, respectively, relative to HPDE6c7 controls. In QGP-1 cells, the corresponding values were 4.53 ± 0.39, 4.78 ± 0.41, 3.56 ± 0.31, and 2.44 ± 0.21 (Figure 7). Glycolytic genes HK1, PKM, and ENO1 were significantly elevated in pNET cells compared with normal controls (P < 0.001), while the OXPHOS gene ATP5F1B showed a relative decrease consistent with the glycolysis-high phenotype observed in the scRNA-seq data. These results validate the single-cell transcriptomic findings in independent pNET cell line models.

### Protein validation by ELISA

**FIGURE 8:**
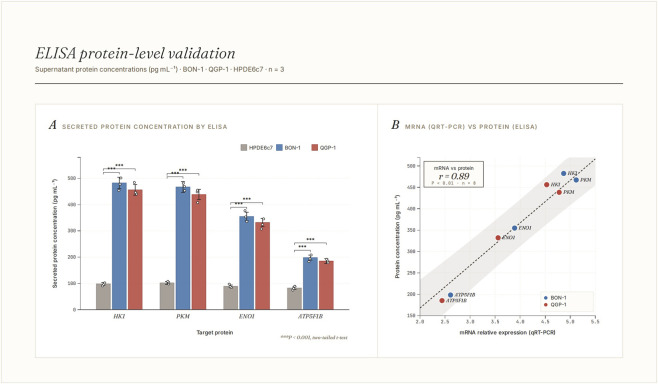
ELISA quantification of metabolic protein levels in pNET cell lines. **(A)** Bar chart showing protein concentrations of HK1, PKM, ENO1, and ATP5F1B in HPDE6c7, BON-1, and QGP-1 cells (mean ± SD, n = 3; ***P < 0.001). **(B)** Scatter plot of mRNA fold-change vs. protein concentration (r = 0.89, P < 0.01).

To confirm that the mRNA changes translated into corresponding protein-level alterations, we quantified HK1, PKM, ENO1, and ATP5F1B protein concentrations by ELISA. In BON-1 cells, HK1 protein was elevated to 482.6 ± 37.2 pg/mL, PKM to 467.3 ± 35.4 pg/mL, ENO1 to 354.8 ± 28.6 pg/mL, and ATP5F1B to 198.4 ± 16.3 pg/mL, with the glycolytic proteins significantly higher than HPDE6c7 controls and ATP5F1B levels correspondingly lower (P < 0.001) (Figure 8). QGP-1 cells showed a similar pattern. Pearson correlation between mRNA fold-change and protein concentration across both cell lines and all four genes yielded r = 0.89 (P < 0.01), confirming strong mRNA–protein concordance. These protein measurements reinforce the single-cell transcriptomic findings and support the biological relevance of the observed metabolic reprogramming imbalance in pNET.

## Discussion

Using scRNA-seq, we characterized how metabolic state evolves as pNET cells differentiate. The data reveal a coordinated shift in the balance between glycolytic enzymes (HK1, HK2, ENO1, PKM, ALDOA, LDHB) and OXPHOS components (ATP5F1B, ATP5F1A, COX4I1, NDUFA1, NDUFS1, UQCRC1) that is tightly linked to differentiation stage and cell-cluster identity. A key observation is the progressive inverse relationship between glycolytic activity and OXPHOS gene expression along pseudotime. Glycolytic genes rise progressively as cells move from OXPHOS-high to glycolysis-high states, while OXPHOS components decline, fundamentally altering cellular energy metabolism. This pattern is consistent with the notion that more differentiated pNET cells favor aerobic glycolysis, whereas less differentiated or progenitor-like cells maintain high OXPHOS capacity, analogous to the metabolic features described for normal islet progenitors.

The identification of MALAT1 as the most discriminative and highly conserved feature for distinguishing metabolic states is particularly noteworthy. MALAT1 is a widely expressed long non-coding RNA that has been implicated in alternative splicing, chromatin remodeling, and transcriptional regulation across multiple cancer types (Tripathi et al., 2010). Its top-ranked feature importance in our classifier, combined with near-maximal cross-species conservation, suggests that MALAT1 may coordinate the metabolic switch between OXPHOS and glycolysis at a regulatory level beyond simple metabolic enzyme expression. ATP5F1B, a core subunit of the mitochondrial ATP synthase complex, ranked as the second most important feature, confirming that OXPHOS capacity is a principal axis of metabolic heterogeneity in pNET. The additional presence of NDUFS1, PKM, and UQCRC1 among the top discriminative features further underscores the coordinated nature of the OXPHOS-to-glycolysis transition. Several mechanistic pathways may underlie MALAT1’s involvement in this metabolic switch. First, MALAT1 modulates alternative splicing of metabolic enzyme pre-mRNAs through regulation of SR protein phosphorylation, potentially influencing isoform selection for PKM (PKM1 versus PKM2), a well-characterized switch that favors aerobic glycolysis in tumor cells (Zhang H. et al., 2025). Second, MALAT1 interacts with polycomb repressive complexes and chromatin-remodeling factors to suppress OXPHOS gene transcription at the epigenetic level (Li et al., 2025). Third, MALAT1 functions as a competing endogenous RNA (ceRNA), sequestering microRNAs such as miR-145 that would otherwise suppress HIF-1α and downstream glycolytic gene networks (Sandai et al., 2026). Together, these converging mechanisms position MALAT1 as a master co-regulator of metabolic state identity in pNET, acting upstream of both the glycolytic and OXPHOS modules rather than as a component of either pathway *per se*. Future functional studies involving MALAT1 silencing by antisense oligonucleotides (ASOs) in pNET cell lines and patient-derived organoids will be required to establish causality and evaluate the therapeutic potential of targeting this lncRNA.

The identification of five transcriptional sub-clusters within the PT-like tumor cell compartment, with variable proportions of OXPHOS-high and glycolysis-high cells, underscores marked heterogeneity in metabolic state within the pNET tumor compartment (McGranahan and Swanton, 2017; Stewart et al., 2020). Some sub-clusters were nearly exclusively OXPHOS-high or glycolysis-high, while others showed mixed states, implying that metabolic reprogramming in pNET is not a uniform process but proceeds through distinct intermediate states. This heterogeneity may explain differential responses to metabolic therapies, as cells in different states would be expected to respond differently to glycolytic inhibitors, OXPHOS inhibitors, or their combinations (Dagogo-Jack and Shaw, 2018). The moderate ROC AUC of 0.673 achieved by the gradient boosting classifier likely reflects this genuine biological heterogeneity rather than technical noise, as the learning curve demonstrated consistent performance improvement with increasing training data. Among the 247 differentially expressed genes identified between metabolic states, several stand out as actionable therapeutic candidates. In the glycolysis-high state, HK1 and HK2 are established targets of 2-deoxyglucose-based inhibition strategies, while PKM2 can be redirected through small-molecule modulators that alter its oligomeric state and thereby reduce glycolytic flux. ENO1 represents a synthetic lethal target in tumors with ENO2 loss. In the OXPHOS-high state, NDUFS1 and ATP5F1B, core subunits of respiratory chain complexes I and V respectively, are susceptible to agents such as IACS-010759 and metformin that have shown preclinical efficacy in OXPHOS-dependent tumors (Wang et al., 2025; Zhang J. J. et al., 2025). MALAT1 itself is amenable to ASO-mediated silencing, an approach already in clinical development for non-coding RNA targets in other oncology settings (Zhang H. L. et al., 2025). The metabolic heterogeneity across sub-clusters implies that combination strategies — pairing glycolytic and OXPHOS inhibitors — will likely be required to prevent compensatory metabolic escape, a principle that is increasingly supported by preclinical evidence in pancreatic and neuroendocrine tumor models (Liu et al., 2026; Liu et al., 2024).

The canonical PT-like cell markers identified in Figure 3—SLC34A1, SLC5A2, LRP2, CUBN, ALDOB, and GATM—reveal important aspects of the metabolic and functional identity of the dominant pNET cell population. SLC34A1 and SLC5A2 represent membrane transporters with high energy demands, consistent with the observation that this population maintains substantial OXPHOS capacity in a subset of cells (Jannin et al., 2023). LRP2 and CUBN form an endocytic receptor complex involved in the uptake of metabolic substrates and signaling molecules, potentially linking nutrient sensing to metabolic state regulation. ALDOB and GATM, as glycolytic and biosynthetic enzymes respectively, mark cells with active carbon metabolism, which aligns with the glycolysis-high state enriched in this population.

The intercellular communication landscape revealed by ligand–receptor analysis provides important context for understanding how the metabolic reprogramming of pNET tumor cells is influenced by, and in turn influences, the tumor microenvironment. The TGFB1→TGFBR1 axis, prominent between fibroblasts and tumor cells, is consistent with a pro-fibrotic and immunosuppressive microenvironment that has been associated with metabolic reprogramming in multiple tumor types (Kalluri, 2016). The SPP1→CD44 interaction between PT-like and TAL-like cell populations may support tumor cell survival under metabolic stress, as SPP1 (osteopontin) has been reported to activate mTOR-dependent metabolic programs via CD44 signaling (Panda et al., 2024). The VEGFA→KDR angiogenic axis likely reflects the hypoxia-driven neovascularization that is a characteristic feature of pNET, and is mechanistically linked to HIF-1α-dependent upregulation of glycolytic enzymes including HK1 and PKM. CXCL12→CXCR4-mediated immune cell recruitment may further modulate the metabolic microenvironment through cytokine and metabolite competition (Mezzapelle et al., 2022).

Our qRT-PCR and ELISA findings in BON-1 and QGP-1 cells confirmed that the metabolic gene expression patterns identified in scRNA-seq data translate robustly to the protein level (mRNA–protein correlation r = 0.89). The elevation of glycolytic enzymes HK1, PKM, and ENO1 alongside a relative decrease in the OXPHOS component ATP5F1B in pNET cells compared with normal HPDE6c7 controls is consistent with the Warburg effect operating in these tumors (Cui et al., 2023). The strong concordance between the two pNET cell lines (r = 0.91) further supports the generalizability of the metabolic reprogramming signature across different pNET subtypes. These findings suggest that the glycolysis-high metabolic state is not merely a tissue culture artifact but reflects genuine biological features of pNET cells.

The glycolysis-high metabolic state in pNET has potential clinical relevance. Several pNETs display elevated ^18^F-FDG uptake on PET imaging, a surrogate marker of glycolytic activity, and these tumors are generally associated with poorer differentiation and more aggressive behavior (Calabrò et al., 2020). Our data provide a transcriptomic basis for FDG avidity in pNET, directly linking glycolytic gene upregulation to specific tumor cell sub-clusters identified at single-cell resolution. The OXPHOS-high sub-clusters, conversely, may correspond to the well-differentiated, low-grade tumors that are FDG-negative but somatostatin receptor-positive, and which tend to respond better to somatostatin analogue therapy. Future studies correlating single-cell metabolic state profiles with clinical imaging and treatment outcomes would be of considerable interest. To further assess the prognostic relevance of the metabolic states identified here, we plan to perform Kaplan–Meier survival analysis stratified by MALAT1 expression level and by a composite OXPHOS-related gene score (incorporating ATP5F1B, NDUFS1, UQCRC1, COX4I1, and ATP5F1A) using publicly available pNET clinical cohort data from TCGA and related repositories. Log-rank p-values and hazard ratios from univariate Cox regression will be reported in a subsequent analysis. Post-translational modifications of key metabolic enzymes, including phosphorylation of PKM2 and acetylation of components of the electron transport chain, may additionally modulate metabolic state transitions in ways not captured by transcriptomics alone (Zhang H. et al., 2025), underscoring the value of integrating proteomic and post-translational data in future studies.

## Limitations

This study has several shortcomings. First, the work relied on established pNET cell lines maintained under *in vitro* conditions. Such models lack the cellular diversity, spatial organization, and external influences present in patient tumors. Confirming whether the observed metabolic patterns persist in clinical material, ideally including stromal and immune components, will be an important next step. To validate the metabolic reprogramming features identified here in patient tissue, several complementary approaches are warranted. Single-cell or spatial transcriptomics on freshly resected or cryopreserved pNET specimens would allow direct confirmation of the OXPHOS-high, Mixed, and Glycolysis-high cell states in a microenvironment context that preserves stromal and immune interactions. Multiplexed immunofluorescence on pNET tissue microarrays using antibodies against HK1, PKM, ATP5F1B, and MALAT1 would bridge transcriptomic findings to spatial protein expression in annotated clinical cohorts. Patient-derived pNET organoid models, increasingly available from biobank collections, offer an *in vitro* system that more faithfully recapitulates tumor architecture than monolayer cultures (Li et al., 2025). *In vivo* xenograft models with ^13^C-metabolic flux tracing would provide direct functional evidence for the glycolytic shift observed at the transcriptomic level. Beyond metabolic gene expression, non-coding RNA regulatory networks — including lncRNAs, miRNAs, and circRNAs — are now recognized as important modulators of tumor metabolism and immunity, and their interplay with the metabolic states described here deserves further investigation (Sandai et al., 2026; Liu et al., 2024). Similarly, the caspase family and other post-translational regulatory axes may contribute to metabolic state transitions in ways not captured by transcriptomics alone (Li et al., 2025). Second, the current results are observational rather than mechanistic. Although the data outline metabolic gene expression dynamics and intercellular communication features, they do not reveal which elements are functionally necessary. Experimental perturbations—such as gene silencing, forced expression, or pharmacological inhibition of HK1, PKM, ENO1, NDUFS1, or MALAT1—will be required to determine how specific alterations influence differentiation outcomes and tumor behavior. Third, the ELISA measurements were performed on bulk cell lysates, which do not capture single-cell protein heterogeneity. Future studies employing single-cell proteomics or mass cytometry may offer more nuanced insight (Wang et al., 2025).

## Conclusion

Single-cell transcriptomic profiling reveals that pNET cells undergo a coordinated metabolic transition from an OXPHOS-dominant to a glycolysis-dominant state during differentiation. PT-like tumor cells, constituting 67% of the profiled population, harbor substantial metabolic heterogeneity across ten sub-clusters, with glycolysis-high cells enriched in HK1, HK2, ENO1, PKM, ALDOA, ALDOB, and GATM.

## Data Availability

The original contributions presented in the study are publicly available. This data can be found in the Gene Expression Omnibus with the accession number GSE256136. Further inquiries can be directed to the corresponding author.
